# An Evolutionary Approach for Identifying Driver Mutations in Colorectal Cancer

**DOI:** 10.1371/journal.pcbi.1004350

**Published:** 2015-09-17

**Authors:** Jasmine Foo, Lin L Liu, Kevin Leder, Markus Riester, Yoh Iwasa, Christoph Lengauer, Franziska Michor

**Affiliations:** 1 Department of Mathematics, University of Minnesota Twin Cities, St. Paul, Minnesota, United States of America; 2 Department of Biostatistics and Computational Biology, Dana-Farber Cancer Institute, and Department of Biostatistics, Harvard T. H. Chan School of Public Health, Boston, Massachusetts, United States of America; 3 Industrial and Systems Engineering, University of Minnesota Twin Cities, St. Paul, Minnesota, United States of America; 4 Department of Biology, Kyushu University, Fukuoka, Japan; 5 Blueprint Medicines, Cambridge, Massachusetts, United States of America; Brown University, UNITED STATES

## Abstract

The traditional view of cancer as a genetic disease that can successfully be treated with drugs targeting mutant onco-proteins has motivated whole-genome sequencing efforts in many human cancer types. However, only a subset of mutations found within the genomic landscape of cancer is likely to provide a fitness advantage to the cell. Distinguishing such “driver” mutations from innocuous “passenger” events is critical for prioritizing the validation of candidate mutations in disease-relevant models. We design a novel statistical index, called the Hitchhiking Index, which reflects the probability that any observed candidate gene is a passenger alteration, given the frequency of alterations in a cross-sectional cancer sample set, and apply it to a mutational data set in colorectal cancer. Our methodology is based upon a population dynamics model of mutation accumulation and selection in colorectal tissue prior to cancer initiation as well as during tumorigenesis. This methodology can be used to aid in the prioritization of candidate mutations for functional validation and contributes to the process of drug discovery.

## Author Summary

Evolutionary dynamic models have been intensively studied to elucidate the process of tumorigenesis. One key aspect of studying tumorigenesis is to distinguish the “driver” mutations providing a fitness advantage to cancer cells against neutral “passenger” or “hitchhiking” mutations. Many statistical models to address this question have been developed. Evolutionary models, however, add another layer of complexity by taking into account the process of mutation accumulation and selection within the tissue. Here we present a novel approach combining both statistical and evolutionary thinking to identify driver mutations in cancer genomes using cross-sectional mutation data. Our method considers the process of mutation accumulation and selection before and during colorectal cancer initiation. This work demonstrates the importance of using evolutionary population dynamic models to study driver events of tumorigenesis.

## Introduction

Cancer cells often harbor hundreds to thousands of genetic changes [1–3]. Many of those changes represent neutral variation that does not influence cancer development; such mutations are called passenger or hitchhiking mutations [1, 4, 5]. A few alterations, however, are essential for driving tumorigenesis. Those changes are known as driver mutations and increase the reproductive fitness of the cancer cell [6–8]. The identification of such mutations is of crucial importance for drug discovery because they represent promising targets for therapeutic intervention.

There is a growing literature of mathematical and statistical approaches to this question [9–18]. In particular, several recent contributions utilized evolutionary models. *Tomasetti et al* [18] investigated a multiphase model of cancer initiation and tumor growth and studied the mutational burden of tumors across various tissue types. They found that the mean number of somatic mutations in tumors of self-renewing tissues is correlated with patient age at diagnosis. In their model, the time of tumor initiation is chosen so the results fit to incidence data. Another evolutionary modeling framework was considered by *Bozic et al* [10]. In this work, the mutation accumulation process during the tumor growth (post-initiation) phase was modeled. The authors proposed a formula relating the number of driver mutations to the total number of mutations in the tumor, and applied this methodology to experimental data to infer the selective advantage conferred by typical somatic mutations. This model was based on the assumption that each driver mutation leads to the same selective advantage over the parent cell fitness. Finally, McFarlane et al [13] argued that passenger mutations can also accumulate albeit weak deleterious effects, eventually resulting in oncogenic phenomena.

Adopting not evolutionary, but data-driven approaches, statisticians and computational biologists have been successful at designing methodology to distinguish driver from passenger mutations. For example, MuSiC [19] compares the mutation rates of genes against the background mutation rates using both the Neymanian likelihood ratio test and the Fisherian combined p-value criterion. *Youn and Simon* [20, 21] further incorporated heterogenous functional impacts of mutations at different nucleotide positions—for instance, missense mutations are considered have less impact than frameshift indels. Later on, using a more ad hoc approach, *Vogelstein et al.* [22] developed a method based on the patterns of mutation frequencies in each gene: they required that > 20% of mutations in a putative cancer gene are located at recurrent positions and are missense, and that > 20% of mutations in a putative tumor suppressor genes are inactivating. MutSigCV [9], improved from the original MutSig method [1], corrected for population and genomic heterogeneity of mutation rates using a high-dimensional approach to decrease the false discovery rate when calling driver mutations based on mutation frequency. Similarly, DrGaP [23] took into account the length of protein-coding regions, transcript isoforms, variation in mutation types, different background mutation rates, redundancy of the genetic code and others and used a likelihood ratio test to obtain significance levels. Moreover, Multi-Dendrix, DriverNet, MuSiC, and MEMo [12, 17, 19, 24–26] were also developed to identify driver pathways using network-based approaches. An integrated meta-analysis using multiple methods can be accessed through DriverDB [27].

Here we describe a computational approach designed to identify alterations that act as drivers during tumorigenesis. We first designed a mathematical model of the evolutionary processes of mutation accumulation both in healthy tissue during the phase prior to tumor initiation, as well as during the clonal expansion phase of the tumor. One novel aspect of our model is the inclusion of flexible mutational fitness distributions during both phases of mutation accumulation; each mutation may confer a random effect on the reproductive fitness of a cell, drawn from a fitness distribution. We tuned this model to specifically describe disease progression in colorectal cancer by combining literature-based estimates of biological parameters with epidemiological data on the incidence of colorectal cancer as well as pre-cursor conditions. We then sought to identify driver mutations by considering a hypothetical neutral mutation at any locus in the genome and following its progression through our evolutionary framework to determine the likelihood of observing this mutation at a detectable frequency in a significant portion of patients with this cancer type. This quantity is called the “Hitchhiking Index” and can be used to reject the hypothesis that any particular candidate mutation is neutral, thus identifying potential driver mutations. Since the likelihood of acquiring each candidate gene mutation may vary across the genome, we stratified mutation rates into three large groups: those with high, intermediate and low mutation rates [9]. By helping to identify mutations that are potential driver mutations during tumorigenesis, this methodology can be used to aid in the prioritization of candidate mutations for functional validation.

### The computational framework

**Fig 1:**
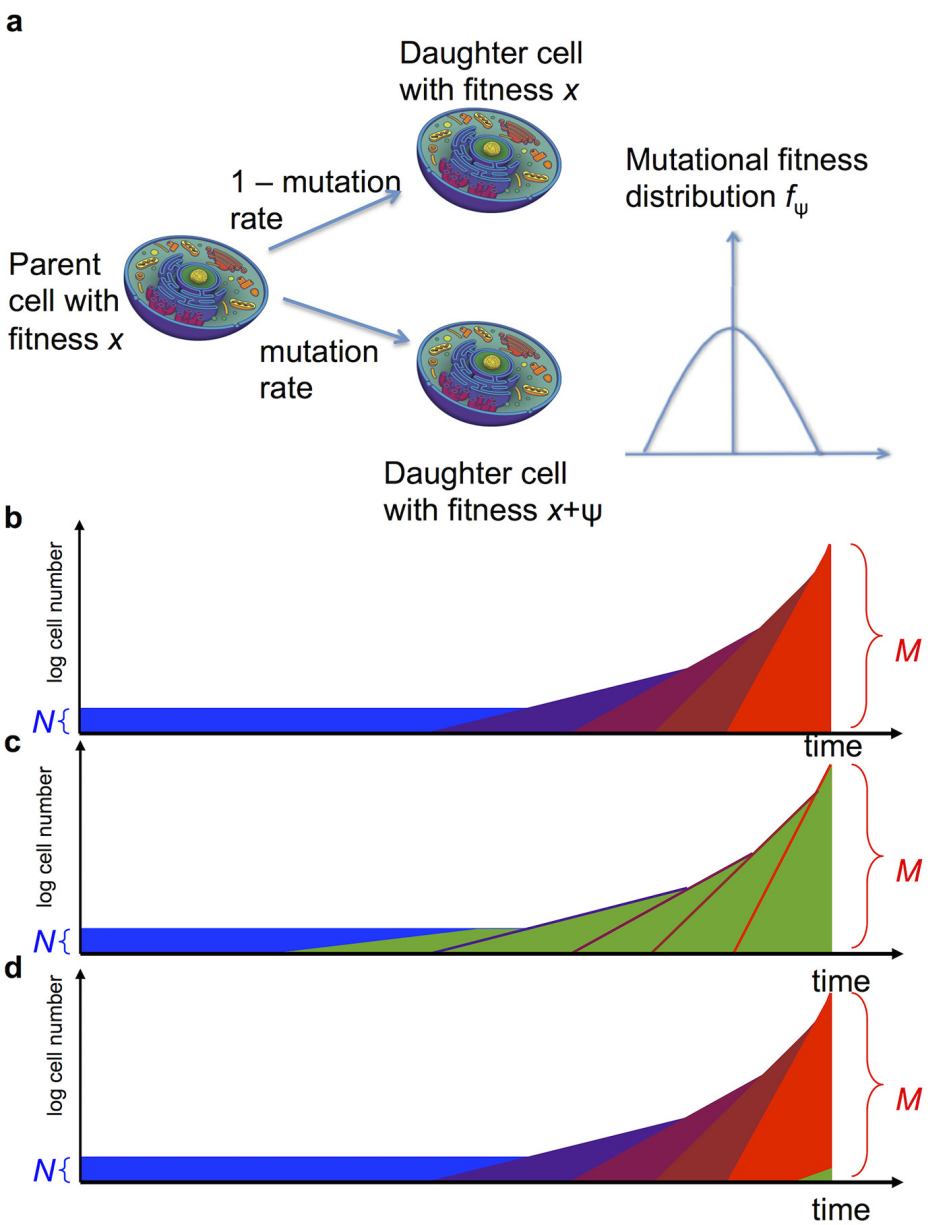
Schematic representation of the model. (a) During each cell division, a mutation might arise which changes the fitness of a daughter cell according to a fitness distribution. (b) Tumor development consists of two phases: the pre-initiation phase, in which the population consists of a constant number of *N* cells (blue), and the post-initiation phase, in which more and more aggressive cell types emerge (shades of purple and red). The tumor is diagnosed when the total population size reaches *M*. A neutral hitchhiker mutation (green) might arise during the pre-initiation phase (c) or during the post-initiation phase (d).

Our computational approach is based upon the evolutionary dynamics of the accumulation of driver and passenger mutations in a population of cells (Fig 1.(a)). There are two phases (Fig 1.(b)): a pre-initiation phase and a clonal expansion phase. During the pre-initiation phase, the first driver mutation has not yet emerged and the population is maintained at a homeostatic cell number. Cells proliferate according to a stochastic process: at each time step, a cell is chosen at random proportional to its fitness to divide, and its offspring replaces another randomly chosen cell. During each cell division, a mutation may emerge with probability *u*. Each mutation confers an additive change to the fitness of the daughter cell; this additive change is chosen from a mutational fitness distribution which is approximated discretely by a mutational kernel *M*_1_. The survival of the resulting mutant clone is dependent on its relative fitness, as well as any subsequent mutations it may accumulate. This phase of the methodology is designed to model the behavior of stem cells within a crypt of the colon.

If a cell in the population has accumulated a sufficiently large fitness to counter the homeostatic mechanisms of the compartment, then the second phase of clonal expansion begins (Fig 1.(b)). The cell number in the tumor is now described by a multi-type stochastic branching process: at each time step, a cell is chosen proportional to fitness to divide (possibly with mutation) or chosen at random to die. This initiated cancer cell carries one or more driver mutations that confer a larger growth than death rate; the population of cells thus grows on average exponentially. Each time a cell in this population divides, a mutation may again arise with probability *u*. Once again, mutations confer an additive change to the fitness of the daughter cell; this additive change is chosen from a mutational fitness distribution which is approximated discretely by a mutational kernel *M*_2_. This mutational kernel is not, in general, the same as the mutational kernel from the pre-initiation phase (*M*_1_), since there is no reason to assume that the mutational fitness distribution in a normal compartment of healthy cells during the pre-initiation phase should be the same as the distribution in a rapidly expanding cancer clone. However, we utilize the same family of mutation kernels, noting that the shape parameters may differ between phases. As mutations accumulate and undergo clonal expansion in the model, the number of cell types grows and the tumor population becomes more heterogeneous. Each additional driver mutation increases the fitness of the cell lineage, such that the rate of expansion accelerates (Fig 1.(b)). Clonal growth continues until the tumor reaches its detection size, *N*_*d*_ (Fig 1.(b)).

We then utilize this underlying evolutionary model to determine the probability *q* that a particular candidate gene is found in a detectable frequency of the tumor, conditioned on the null hypothesis that alterations of this gene are selectively neutral. This probability *q* is then used to derive the probability of obtaining the observed frequency of alterations in the sample set, given the null hypothesis. More specifically, when sampling tumors from *Y* patients, we calculate the “Hitchhiking Index”, which specifies the probability of detecting a certain mutation in at least *α*% of *Y* patients, as

$$H=\sum_{k=\lceil \alpha Y\rceil }^{Y}P\left(X=k\right),$$

where *X* is binomially distributed as *Binom*(*Y*,*q*) and ⌈*αY*⌉ is the smallest integer greater than *αY*. This index provides a tool for rejecting the null hypothesis. If for example *H* < 0.001 for a given observed *α* and *Y*, we can reject the assumption of neutrality of a mutations in a given gene of interest.

The parameters of this model (e.g. cell turnover rates and mutation kernels in both phases, population size during homeostasis and at detection) must be specifically tuned for each cancer and sample type. In addition to the evolutionary processes within the tissue, the value of the Hitchhiking Index also depends upon the relative mutation rate of each candidate gene, detection sensitivity of the sample set, and the observed alteration frequency in the sample set. Note that the Hitchhiking Index provides a method for rejecting the null assumption of neutrality; however, failure to reject this assumption does not necessarily imply that the gene is neutral.

### The pre-initiation phase

Let us provide further details of underlying mathematical framework that models the pre-initiation phase, which was first introduced in [28]. During this phase we consider a small population of cells of constant size *N* which describes a homeostatic compartment of cells at risk for accumulating mutations leading to cancer initiation, described by a multi-type Moran process [29]. Each cell on average divides every *D* days. Thus, at rate *N*/*D* (i.e. time between events are i.i.d exponential random variables with mean *D*/*N*), the process undergoes division events. During each event, a cell is chosen at random to die, and proportional to its fitness an individual is chosen to reproduce. Specifically, if there is a single cell with fitness *s* and *N*−1 cells with fitness 1, then the cell with fitness *s* is chosen to reproduce with probability *s*/(*s* + (*N*−1)). During each cell division event, an (epi)genetic alteration may occur with probability *u* < < 1; thus alterations arise in the compartment of cells at rate *Nu*.

The fitness effects of individual alterations are random variates drawn from a mutational fitness landscape governed by the mutation kernel *M*_1_(⋅, ⋅). Here *M*_1_(*x*,*y*) represents the probability that a cell with fitness *x* produces a daughter cell with fitness *y* (i.e. $M\left(x,y\right)=f_{\psi }^{x}\left(y-x\right)$). If *y* > *x*, then the fitness of the daughter cell is advantageous as compared to the fitness of its parent cell; if *y* < *x*, it is disadvantageous, and if *y* = *x*, it is neutral. The type space is discretized into fitness bins to aid in computational tractability, and thus the kernel *M* is a finite-state transition matrix. In this work we utilize a general family of mutation kernels that have exponentially decaying tails on the positive and negative sides with shape parameters *α* and *β*, respectively. Note that *α* = *β* = 0 represents the uniform distribution case, and for *α*,*β* > 0 the mutational fitness distribution has a mode at 0 (neutral mutations). This process continues for as long as the fitness of all cells is within the homeostatic range [*a*,*b*] for *a* = 1−1/*N* and *b* = 1+1/*N*; these values were chosen since they signify the boundaries for neutral evolution [30]. Also note all sample paths will result in cancer initiation prior to death.

In the event that a cell with a sufficiently large fitness emerges, it can escape homeostatic mechanisms in the compartment and initiate clonal expansion. It has been shown that when 3*Nu*(log*N* + *γ*) ≪ 1, where *γ* is the Euler-Mascheroni constant, the time between mutational events is much larger than the time it takes for a mutation to take over or go extinct in a population of cells (see, e.g. [28]). In the application of colorectal cancer considered in this work, this condition holds, supported from the parameterization discussed in the section *Model parameterization*. Therefore, on the timescale of interest, the population moves between various homogenous states and we can approximate this process by a Markov process *Z*(⋅), where *Z*(*t*) represents the fitness of the homogeneous compartment at time *t*. The process *Z* jumps whenever a cell harboring a novel non-neutral mutation reaches fixation in the compartment, and takes values in the space of all possible fitness values dictated by the fitness landscape. This process closely approximates the behavior of cellular fitness values in a small compartment for the vast majority of time.

Given that a mutation of fitness *y* arises in a population of cells with fitness *x*, the probability that the mutation takes over the population is given by *r*_*x*,*y*_ = (1−*x*/*y*)/(1−(*x*/*y*)^*N*^). By symmetry we have *r*_*x*,*x*_ = 1/*N*. With this information, we define the intensity matrix for the Markov process *Z*, denoted by *Q* = *Q*(*x*,*y*),

$$Q\left(x,y\right)=\{\begin{array}{c} ur_{x,y}M_{1}\left(x,y\right)N/D,\;y<b\\ uNM_{1}\left(x,y\right)/D,\;y\geq b, \end{array}.$$

Mutational events transforming a cell with fitness *x* to a cell with fitness *y* occur at rate *uM*(*x*,*y*)*N*/*D*, and of those a fraction *r*_*x*,*y*_ reach fixation (i.e. 100% frequency) in the entire population. At the lower end of the fitness range *a*, there is a reflecting boundary such that any fitness below *a* is immediately replaced by a cell with fitness *a*; this behavior is implemented in the mutation kernel *M*. By definition, we have *Q*(*x*,*x*) = −∑_*y* ≠ *x*_ *Q*(*x*,*y*), and the states *x* ≥ *b* are absorbing so *Q*(*x*,*y*) = 0 for all *y* and *x* ≥ *b*. The summation is over all *y* in the discrete fitness space.

The process *Z* represents the dynamics of the fitness of a healthy compartment of cells over time; this process is then conditioned upon the event that cancer initiation occurs during a human lifetime [28]. This is achieved by creating an additional lifetime process, *L*, that is run simultaneously with *Z* in a second dimension. The lifetime process has a single absorbing state representing death of the patient, and transition rates between intermediate stages are tuned using mortality statistics in the United States. Details of this tuning process can be found in [28]. We are then interested in the joint process (*Z*,*L*) conditioned on *Z* hitting its absorbing state (fitness greater than *b*) prior to *L* hitting its absorbing state (death). This set of sample paths describes the cancer initiation paths which may lead to tumor diagnosis prior to death.

To consider the fate of a particular candidate mutation ‘A’ in our data set, suppose that this mutation arises with probability *u*_0_ per cell division. Under our null hypothesis, we assume mutation A is neutral and confers no selective advantage/disadvantage; thus its presence does not alter the evolutionary outcome of the sample path. Conditioned on initiation prior to death, we compute the probability of mutation A arising in the initiating cancer cell. To do this, we analyze the amount of time the (*Z*,*L*) process spends in each state of the two-dimensional state space. Details of this derivation are provided in the Methods.

### The clonal expansion phase

Once a cell in the pre-initiation phase has acquired a fitness value greater than *b*, the second clonal expansion phase of the model commences. This phase is modeled by a continuous time multi-type birth and death process, initiated by the cell from the pre-initiation phase that has accumulated a sufficiently large fitness to break free from the Moran process and initiate clonal expansion. The initial branching process has birth rate *b* and death rate *d*, where *b* > *d*. During this expansion phase, each time a cell divides, it has a probability *u* of mutating and selecting a new random birth rate from a fitness distribution, and probability *u*_0_ of obtaining the specific candidate mutation A without any change in birth rate. The birth rate of a mutated daughter cell is the parental fitness plus a random variable selected from a distribution specified by the mutation kernel *M*_2_. The type space is once again discretized into fitness bins to aid in computational tractability, and the kernel *M*_2_ is a finite-state transition matrix, accordingly.

### Simulation methodology

We then aim to determine the probability that mutation A is present in a significant fraction of the final population size at detection, *N*_*d*_, conditional upon the event that the expansion process was initiated and reached detection size during a human lifetime. To study this event we first utilized analytical calculations to determine the probability of tumor initiation prior to death, in the first phase of the model, by solving the linear system described in [28] using a biconjugate gradient stabilized method. We next performed event-driven Monte-Carlo simulations of the two-dimensional Markov process describing the fitness of the crypt and the lifetime state conditional on initiation prior to death [28]. To account for the fact that sample paths of interest may be rare, the probability of initiation calculated in the previous stem was used to perform a *Doob h-transform* of the process (*Z*,*L*) conditioned on initiation prior to death. Thus we only simulated sample paths that lead to initiation, saving computational time.

At the time of initiation for each sample path, the time of initiation as well as the status of mutation A in the initiating cell is recorded. These initial conditions are used to begin a stochastic simulation of the clonal expansion process. During each event in the simulation, a cell is chosen to divide based on its relative fitness and abundance. During each cell division, a mutation occurs with probability *u* and the outcome of that mutation is selected from the mutation kernel *M*_*c*_. The tagged mutation A arises with probability *u*_0_ ≪ *u*. Naturally, a certain fraction of paths in the expansion phase die out at early times due to stochastic fluctuations. For the remaining paths, the simulation is halted when the total population hits the detection size *N*_*d*_, and then the abundance of mutation A in the total population of tumor cells is recorded.

## Results

### Application to colorectal cancer

To apply this framework to analyze genomic data from any specific cancer type, the main challenge is to determine the probability *q* that a particular mutation of interest, mutation A, arises during the clonal expansion phase and eventually makes up a significant fraction of the final population size, *M*. This outcome can occur via two possible scenarios: (1) mutation A arises and reaches 100% frequency during the pre-initiation phase, so that all cells of the resulting tumor have mutation A (Fig 1.(c)), and (2) mutation A is not present in the initiating cell but arises during clonal expansion (Fig 1.(d)). Recognizing these two mutually exclusive possibilities suggests an interesting question: which is the more likely path out of these two scenarios? The answer to this question depends on the parameters of the evolutionary model, which might vary from cancer type to cancer type. We investigated colorectal cancer in particular, through the approach outlined in the following.

#### Model parameterization

The parameters of our model can be divided into two sets according to which phase (pre-initiation or clonal expansion) they belong to. Table 1 shows the parameters involved during the pre-initiation and clonal expansion phases.

**Table 1:** Parameters of the evolutionary model.

Parameter	Definition	Phase
*N*	number of cells at risk for accumulating oncogenic mutations	pre-initiation
*D*	division rate of cells at risk for accumulating oncogenic mutations	pre-initiation
*u*	overall mutation probability per cell division	both
*α*_1_, *β*_1_	shape parameters of mutational fitness kernel	pre-initiation
*b*, *d*	birth/death rate of cell initiating clonal expansion	clonal expansion
*α*_2_, *β*_2_	shape parameters of mutational fitness kernel	clonal expansion
*N*_*d*_	number of cells at time of detection	clonal expansion

We estimated these parameters in colorectal cancer using a combination of biological knowledge and clinical incidence data. A normal human colon contains approximately 15 million crypts, and these crypts contain approximately 10 stem cells each that divide on average once every 7 days [10]. As such, we estimate the population size during the pre-initiation phase to be *N* = 10 and set the baseline birth rate of healthy stem cells *D* to be once every 7 days. The mutation probability per cell division *u* is estimated to be 3⋅10^−3^; this follows from an estimate in [31]: the mutation rate per base pair per cell division is roughly from 10^−10^ to 10^−11^, and the size of the human exome is 3×10^7^, and thus *u* = 10^−10^⋅3⋅10^7^ = 3⋅10^−3^. The diameter of tumors at diagnosis is around 1–10 centimeters and for each 1*cm* ^3^ volume there are approximately 10^9^ cells [32]. It is commonly thought that only a small subset of the tumor cells are capable of propagating the entire tumor cell population over time, and that these so-called cancer ‘stem’ cells comprise perhaps 1 in 10000 cells within the tumor [33]; thus the tumor detection size *N*_*d*_ is set as 1 million cancer (stem) cells. The remaining parameters to set are the mutational fitness kernels as well as the birth/death rate of the cell initiating clonal expansion. These parameters are estimated using epidemiological data as outlined below.

The SEER database (seer.cancer.gov) states that the median age of colorectal cancer diagnosis in the US is approximately 69 yrs and the lifetime risk of colon cancer is around 4.8%. A significant percentage of the population develops aberrant crypt foci (analogous to an initiated crypt) during their lifetime, although exact incidences of this condition are unknown. We thus suppose that the probability of initiation of at least one crypt within the entire colon should be of the order of magnitude of 10–20 percent. Using this data, we then estimated the mutational fitness distribution for each phase and initial expansion rate of the cell population to recapitulate these incidence estimates. A simple computational search of the parameter space for *α*_1_,*β*_1_,*α*_2_,*β*_2_,*b*,*d* was conducted to find a set of parameters that lead to model behaviors closely matching the three incidence data points discussed; however, there are multiple parameter sets that can yield good matches. Thus, we tested the sensitivity of our model predictions to these parameters in the model (Methods).

**Fig 2:**
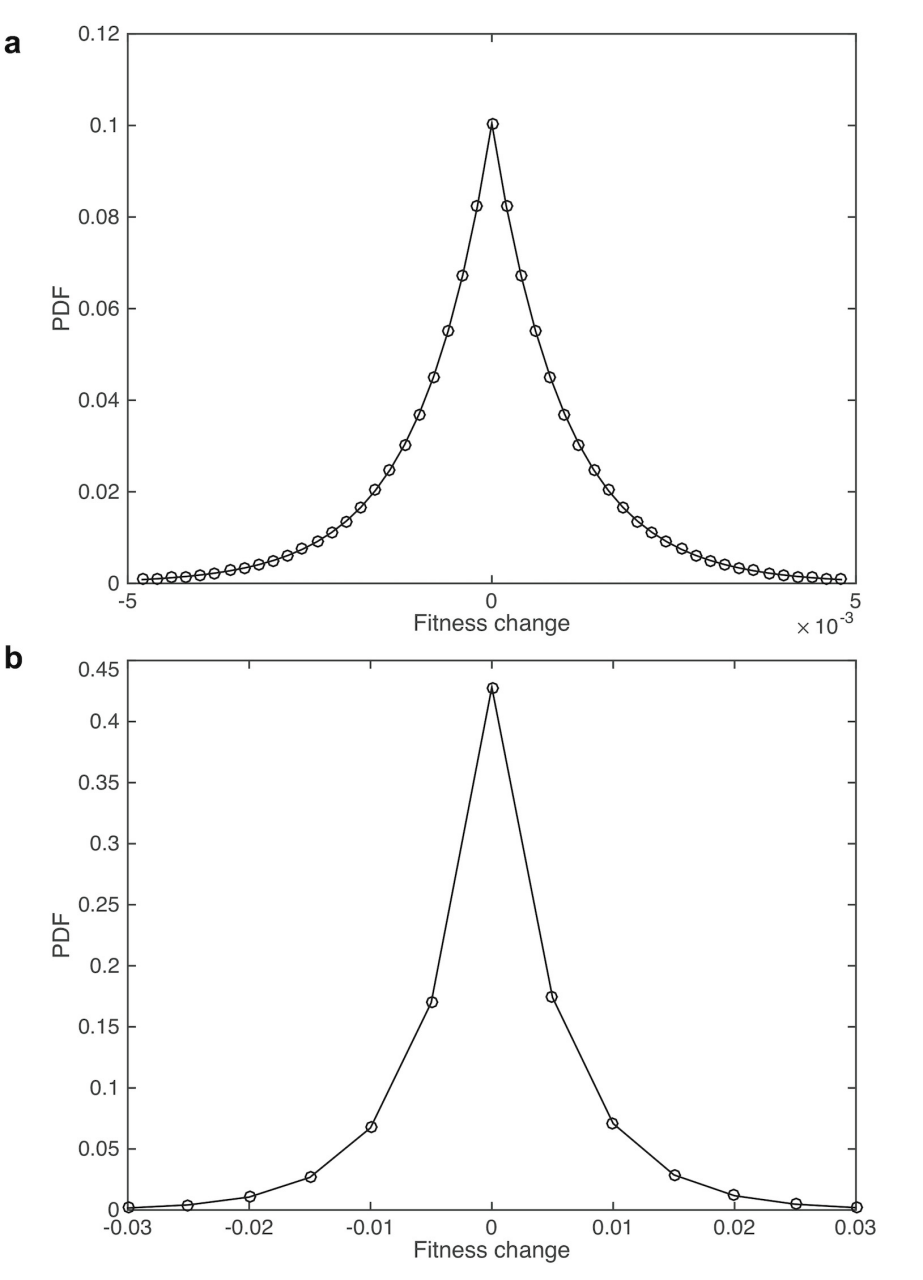
Probability density function of mutational fitness change in the pre-initiation phase (a) and clonal expansion phase (b). Parameterization for this example: in the pre-initiation phase, the decay rate parameter is 180, and for the expansion phase the decay rate is 1000.

Using this procedure, our estimated mutational fitness distributions are shown in Fig 2. (parameters *α*_1_ = *β*_1_ = 180,*α*_2_ = *β*_2_ = 1000) and the estimated initial birth and death rates in the expansion phase are 0.004 and 0.001 *days* ^−1^, respectively. To illustrate the match or the resulting predictions with incidence data, we computed the probability of initiation from a single crypt during the Moran phase to be 1.15×10^−8^. Since there are approximately 15 million crypts in the human colon, this leads to an overall incidence of aberrant crypt foci of approximately 17 percent. Once initiation occurs, the probability of reaching tumor detection size between the time of initiation and the end of the lifetime is 0.28. Therefore, the event that any single crypt initiates and leads to a detectable cancer has probability 1.15×10^−8^×0.28 = 3.23×10^−9^. Then, if considering a binomial random variable with parameters 15 million (number of crypts) and success probability 3.23×10^−9^, we obtained the lifetime risk of cancer to be approximately 4.85%. We also found computationally that the average age of diagnosis is between 65 and 70 years, and that for these cancers the age at the time of cancer initiation is around 55 to 60 years. We performed extensive sensitivity analyses demonstrating that, when varying the fitness distribution with the restriction that the model predictions be consistent with incidence data, the changes to the Hitchhiking index are negligible (Methods).

Finally, the parameter *u*_0_ represents the probability that our candidate neutral mutation arises during each cell division. To take into consideration the local variation across the genome of the mutation rate [9, 34], we first calculated the mutation rate for each genomic locus using the recently published analytical methodology MutSigCV [9]. This method estimates differential mutation rates between genes on the basis of DNA replication timing, chromatin state, and transcription activity level. We then categorized all genes into three different categories: the “high mutation rate” group, “intermediate mutation rate” group, and “low mutation rate” group based on K-means clustering (*K* = 3) of the mutation rate of all genes, where the mutation rate is defined as $\frac{n_{silent}}{N_{silent}}$ from the standard MugSigCV output. Then the median mutation rates were calculated for all three categories. Next, the ratios of “high” vs.“intermediate” and“low” vs “intermediate” categories, *r*_1_ and *r*_2_, were applied to calculate the mutation rate per cell division by setting *u*_*intermediate*_ = *u*_0_, *u*_*high*_ = *u*_*intermediate*_⋅*r*_1_, and *u*_*low*_ = *u*_*intermediate*_/*r*_2_. Note that the baseline rate *u*_0_ is not based on the mutation rates inferred from cancer patients, but are determined as outlined above. However, the ratios between high, intermediate and low mutation rate categories are inferred from cancer tissues. Unfortunately, no accurate estimates are available for these quantities in normal tissue in order to determine these ratios for pre-initiation stage of the model, so the cancer-derived rates are used for both phases of the model.

#### Determining the Hitchhiking Index for each candidate gene

**Fig 3:**
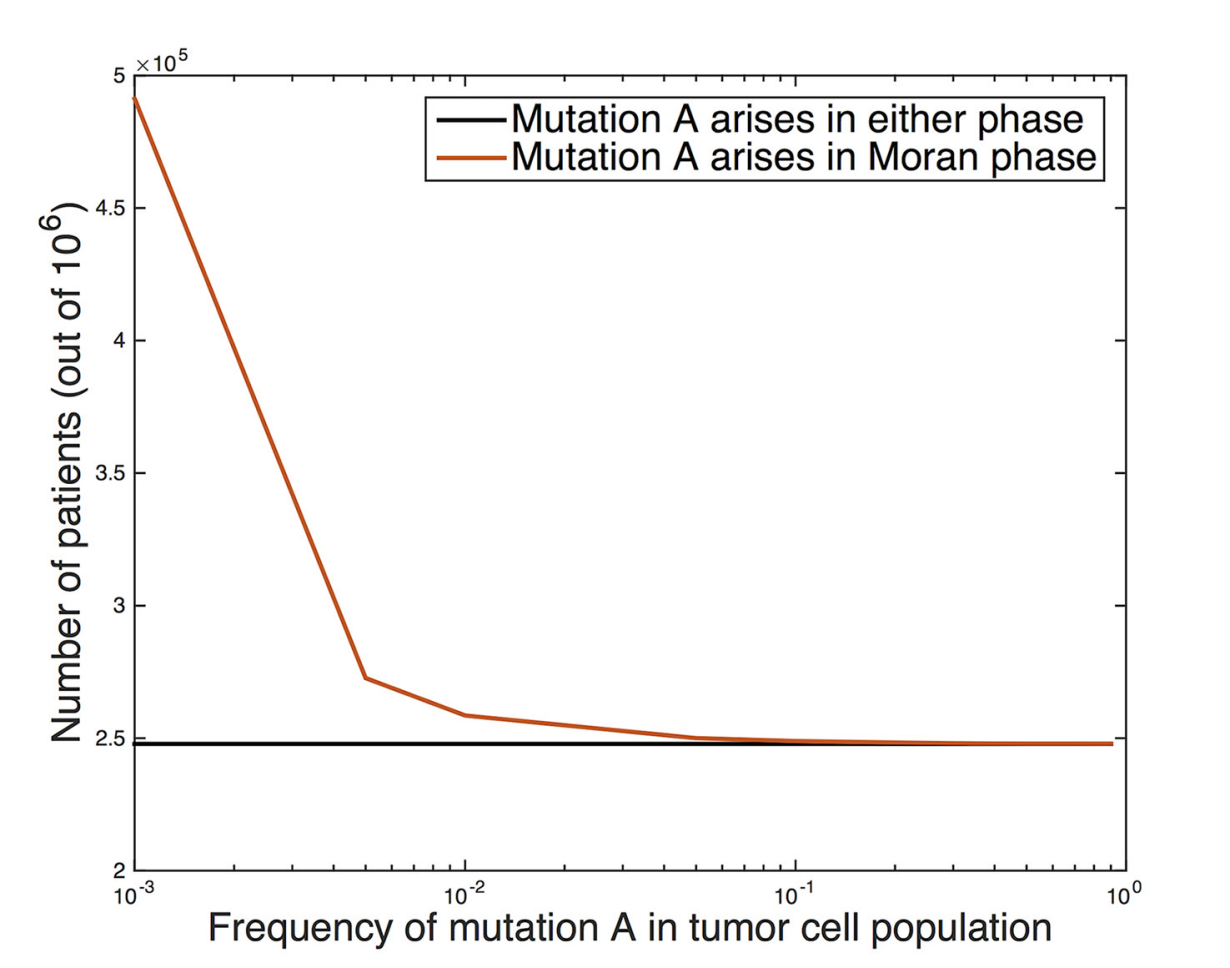
Number of patients out of 1 million samples in which cells with candidate mutation ‘A’ make up a threshold (x-axis) frequency in the ending tumor cell population. (Black) all patients, (red) patients in which mutation A arose and reached fixation (i.e. 100% frequency) during the pre-initiation phase.

We then applied our methodology to determine a frequency cut-off for passenger mutations by considering cross-sectional data from colorectal cancer patients. We simulated the model using the parameters outlined above for 1 million samples to determine the probability *q* that mutation A arises in more than x % of cells in the tumor at detection, for a spectrum of mutation rate levels. Fig 3. shows the number of samples (out of 1 million) in which the cell population harboring the mutation of interest comprises more than a certain percentage (i.e detection threshold) of the final population. The minimum detection threshold percentage is varied on the x-axis. For example, Fig 3. shows that if the mutation is detectable at a frequency of 0.01 and higher, it is expected to be observed with probability *q* = 0.26). For mutation rates of 10^−5^ and 10^−4^ per gene, assuming that the mutation is detectable at a frequency of 0.01 and higher, we obtain *q* = 0.027 and 0.26, respectively. Remarkably, in the vast majority of the time, if the mutation is present in a significant percentage of the tumor (e.g. 10 percent or higher), it arose during the pre-initiation phase.

**Fig 4:**
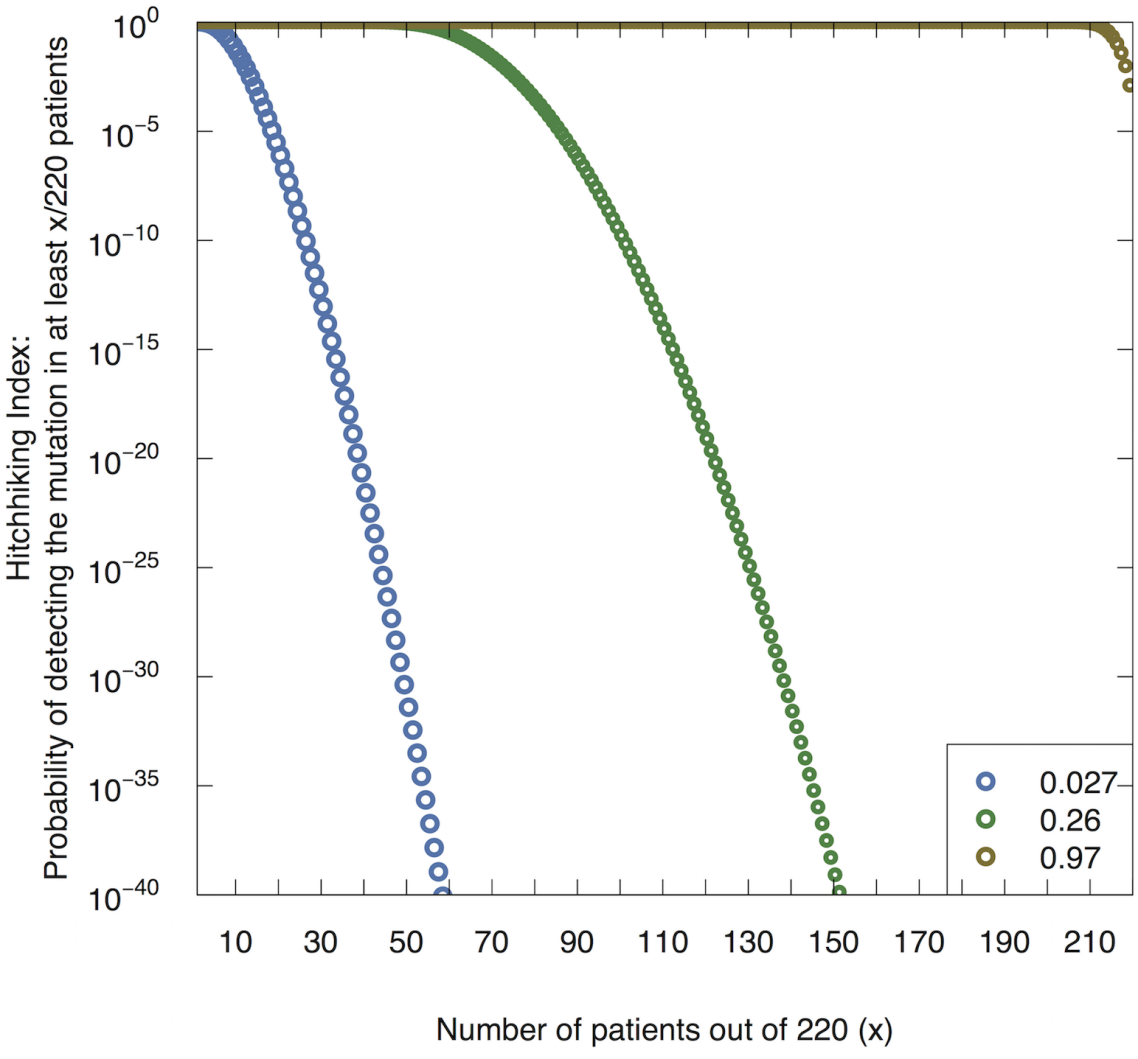
The Hitchhiking Index. Probability of detecting the passenger mutation in x out of 220 patients for a variety of values *q*, which represents the probability that the candidate mutation ‘A’ is present in a sufficiently large fraction of the final population size to be detected.

We then applied our methodology to analyze possible driver genes in colorectal tumors based on data from The Cancer Genome Atlas (TCGA) [35]. In particular, we utilized the calculated *q* values for each mutation rate to determine the frequency threshold for rejecting the hypothesis that a particular candidate genetic mutation is a passenger mutation, by calculating the Hitchhiking Index for each gene family. Fig 4. shows the Hitchhiking Index, i.e. the probability of observing at least *x*% of patients with a candidate mutation in the set of 220 TCGA patients, for several different *q* values. For example, if we are considering a candidate mutation whose background mutation rate is 10^−5^, our calculated *q* value is 0.027. Fig 4. then shows that if the mutation is neutral, the probability of observing at least 19 patients with alterations in this gene is less than 10^−5^. Thus we may use the Hitchhiking Index to set a threshold for rejecting the hypothesis (or alternatively determine a p-value) that a particular candidate gene mutation is a passenger mutation.

Using this methodology with a cutoff of hitchhiking index 10^−11^, we identified a total of 43 genes to be drivers of colorectal tumorigenesis (S1 Dataset). Our method is able to identify the known driver genes for colorectal cancer; additionally, we also identified some driver genes not found by the MutSigCV study, such as COL12A1, MLL2, FAT4 and ARID1A. There exists literature supporting the crucial role of these genes in the development of colon cancer: for example, COL12A1 has been reported to be expressed in colon cancer patients [36]; FAT4 has been shown to have increased recurrent mutation in colorectal cancer [37]; MLL2 is known to have altered expression in colon cancer as well [38]. Finally, ARID1A, a chromatin remodeling gene, has also been reported to be mutated in colon cancer [39].

We then performed a power analysis to provide some guidance about how many patients would need to be profiled in order to obtain a more complete list of colorectal driver genes. Out of 220 patients in the data, we randomly selected 10, 20, ⋯,210 subsamples, performed all the above analyses on the subsamples, and identified the number of driver genes using the same cutoffs. The number of driver genes identified using this approach is shown in S1 Fig. We found that as the number of patients increases, the number of genes identified as driver genes by our approach increases linearly; thus, according to our methodology, a large sample size of profiled patients would be required for the size of the identified driver gene set to level off.

## Discussion

In this work we have developed a novel methodology to identify driver mutations from cross-sectional tumor sequencing data, based on an evolutionary model of tumorigenesis. We developed the ‘Hitchhiking Index,’ which represents the probability of observing alterations in a particular gene in a certain fraction of the patient sample set, under the null assumption that the gene is not a cancer driver. This index takes into account the impact of a number of important parameters on the statistical power of the conclusion: the sample detection threshold (sensitivity of the sequencing method), patient sample size, and variable mutation rates across the genome. The underlying evolutionary model is designed and parameterized for colorectal tumorigenesis, but can be generalized to other cancer types. Here we have not incorporated full pathway information of the gene of interest, but the model can easily be adapted to group genes together into pathways and analyze selection dynamics at the pathway level instead of at the individual gene level. The Hitchhiking index is calculated for any particular candidate gene, using the observed patient sequencing data, and can be used to identify candidate genes as potential drivers.

We applied our methodology to analyze TCGA data for colorectal cancer, considering heterogeneous mutation rates measured per cell division which are inferred from baseline mutation rate estimates and relative changes from that rate across the genome as determined by MutSigCV [9]. We built upon MutSigCV by incorporating heterogeneous mutation rate estimations into our model: (1) We specify an underlying evolutionary dynamic model to describe the processes generating mutations to calculate the probability of a mutation being a driver event; and (2) by controlling for the bias introduced by DNA replication timing, gene expression and higher-order chromatin structure, we infer the relative mutation rate per cell division compared to the cross-sectional mutation rate. Remarkably, we found that any gene that is mutated in at least 10% of cells in the tumor is most likely to have arisen prior to clonal expansion of an initiated cell clone. Utilizing the Hitchhiking Index analysis, we obtained a list of 43 genes identified as potential drivers. In comparison to a recent analysis utilizing MutSigCV [9], our methodology identified other colorectal cancer related genes such as COL12A1, MLL2, FAT4m and ARID1A. Recent studies support the crucial role of these genes in the development of colon cancer [36–39].

One caveat to our approach is that it is unclear how to choose the threshold Hitchhiking Index value; similar to a statistical p-value, the choice of threshold at which to reject the null hypothesis is largely a matter of choice. Since this index depends upon the sample size and detection sensitivity of the method, it would be difficult to compare absolute values of the Hitchhiking Index across different sample sets. Note that if the Hitchhiking Index for a particular gene is above the rejection threshold, we do not conclude that the gene is necessarily a passenger—our methodology provides only the probability of observing the data, conditioned on the assumption of neutrality. Also, note we used the same *u* and *u*_0_ in both phases. While the precise values are unknown, it is possible that the mutation rates can be higher in the latter phase, since it is possible that the initiating mutation causes an increase in the mutation rate itself, e.g., a mutation that reduces the effectiveness of DNA repair. This scenario has the potential to alter the creation rate of passenger mutations and will be the topic of future investigations. As such, our current work excludes consideration of colorectal cancers with microsatellite instability, a deficiency of the mismatch repair (MMR) pathway that leads to increased point mutation rates across the genome. Additionally, hereditary forms of colorectal cancer are also not explicitly considered and will be investigated in future work. Furthermore, the presented model does not include all possibilities for alternative initiation mechanisms. Such a model would not be very useful since it would address mutually exclusive evolutionary trajectories; instead, we have presented one possible model of the evolutionary process leading to tumorigenesis. A novel feature of this model is the formulation of the initiation event as an accumulation of a sufficiently large fitness advantage in the initiating cell through a flexible series of mutational events rather than a specific set or number of hits. Because of this flexibility, a multitude of mutational pathways can lead to initiation in our model, and in particular it can be used to consider the situation in which each of these events is disruption of a particular pathway. The approach can also modified to incorporate the more traditional view that a specific set of hits is required to initiate cancer (by specifying instead a discrete distribution for the mutational fitness landscape) and making this landscape dependent on the current mutation status.

Here, we have utilized a specific, tunable evolutionary model of mutation accumulation in cancer to develop a novel statistical test for identifying driver mutations from cross-sectional genomic data of cancer sample sets. We have opted for a somewhat more flexible approach to modeling the process of mutation accumulation and initiation. For instance, we have considered mutational heterogeneity in a coarse manner, by grouping genes into three different categories with different baseline mutation rates per cell division. A more complete model could in principle use different baseline mutation rates for each categories of DNA replication timing, gene expressions and other genomic features, even including the difference between transitions and transversions [15, 40]. In contrast to the model by *Tomasetti et al* [18], in which all mutations prior to initiation are considered to be selectively neutral and the time of tumor initiation is set by epidemiological data, here we have assumed that mutations conferring random fitness advantages can arise during the constant population size phase, and that tumor initiation occurs as a result of accumulating sufficiently many advantageous mutations to escape homeostasis. Consequently, in our model the timing of cancer initiation is random, and correlated with the process of mutation accumulation. We have carried the same modeling framework through to the tumor growth phase, in which cells may accumulate mutations conferring a spectrum of fitness changes. Consequently, in contrast to the model by *Bozic et al* [10], we assume that driver mutations may be variable in number and lead to variable fitness effects and that tumors may alternatively have many drivers with small selective advantage or a few drivers with large selective advantages. These differing modeling choices reflect a rich set of hypotheses about the underlying evolutionary dynamics of mutation accumulation in cancer; more modeling and experimental effort is needed to investigate the perspectives and relative strengths of these and many other models. Several important conclusions, however, seem robust: first, mathematical analyses of the evolutionary processes in cancer suggest that the majority of mutations found in tumor sequencing efforts arise prior to cancer initiation; and second, mathematical frameworks of evolution and mutation accumulation in cancer can be exploited to extract important biological information from genomic sequencing data.

## Methods

### Probability that mutation A is present at the start of clonal expansion

Let *m*(*t*) denote the number of cells carrying mutation A that are present in the compartment at time *t*. We define the stopping times *τ* = inf{*t* ≥ 0 : *Z*(*t*) ≥ *b*} and *σ* = inf{*t* ≥ 0 : *L*(*t*) = *d*}. We are interested in finding

$$\mu_{A}\left(x,r\right)=P_{\left(x,r\right)}[m(\tau )>0|\tau <\sigma ],$$ (1)

where *μ*_*A*_(*x*,*r*) represents the probability that mutation A is present in the compartment at the time of initiation starting from state (*x*,*r*). Between jumps of the two-dimensional process (*Z*,*L*), a random number of neutral mutations can reach fixation within the compartment of cells. Let *T*_*j*_ and *T*_*j*+1_ be the jump times of (*Z*,*L*), and for simplicity denote (*Z*(*T*_*j*_),*L*(*T*_*j*_)) = *X*_*j*_. During the transition from *X*_*j*_ to *X*_*j*+1_, the compartment can accumulate *Y*_*j*_(*X*_*j*_) neutral mutations. Define

$$\rho \left(x,r\right)=\frac{uM_{1}\left(x,x\right)/D}{uM_{1}\left(x,x\right)/D-Q\left(x,x\right)-S}.$$

The numerator represents the rate at which neutral mutations which eventually reach fixation arise within the compartment, and the denominator represents the total rate at which fixating mutations arrive and the time process changes. With this definition, *Y*_*j*_(*x*,*r*) is distributed like a geometric random variable with

$$\text{Prob}\left(Y_{j}\left(x,r\right)=n\right)=\eta {\left(x,r\right)}^{n}\;(1-\eta (x,r)),$$

which gives

$$\text{Prob}(Y_{j}\left(x,r\right)>0)=\rho \left(x,r\right).$$

By conditioning on the first step we can see that *μ*_*A*_(⋅,⋅) satisfies the following equation for each possible fitness *x* in [*a*,*b*],

$$\mu_{A}\left(x,r\right)=\rho \left(x,r\right)+\sum_{y}\frac{Q_{r}\left(x,y\right)\mu_{A}\left(y,r\right)}{uM_{1}\left(x,x\right)/D-Q\left(x,x\right)-s}-\frac{Q_{x}\left(r,r+1\right)\mu \left(x,r+1\right)}{uM_{1}\left(x,x\right)/D-Q\left(x,x\right)-S}\;,$$

where we note that *μ*_*A*_(*x*,*r*) = 0 for those fitnesses that lie outside of [*a*,*b*]. Therefore we can find *μ*_*A*_(⋅,⋅) by solving the linear system.

### Sensitivity analysis

We tested the sensitivity of the model predictions to varying parameters. First, we studied the sensitivity to the parameters for which we have no experimental data-based estimates: the shape parameters of mutational fitness distribution and the birth and death rates of the initiating cell. We also investigated the sensitivity to the background mutation rate *u*, and in particular studied the impact of an increased mutation rate during the clonal expansion phase. For all of these sensitivity analyses we confined our parameter variation to the ranges in which the model is consistent with the population-level epidemiological data. In particular, we required that there is a significant incidence of aberrant crypt foci over a lifetime (10–90 percent of the population), that the average age at diagnosis is between 70–79 yrs, and that the lifetime risk of colon cancer is around 6–10%.

### Sensitivity to *α*_1_ and *β*_1_

**Fig 5:**
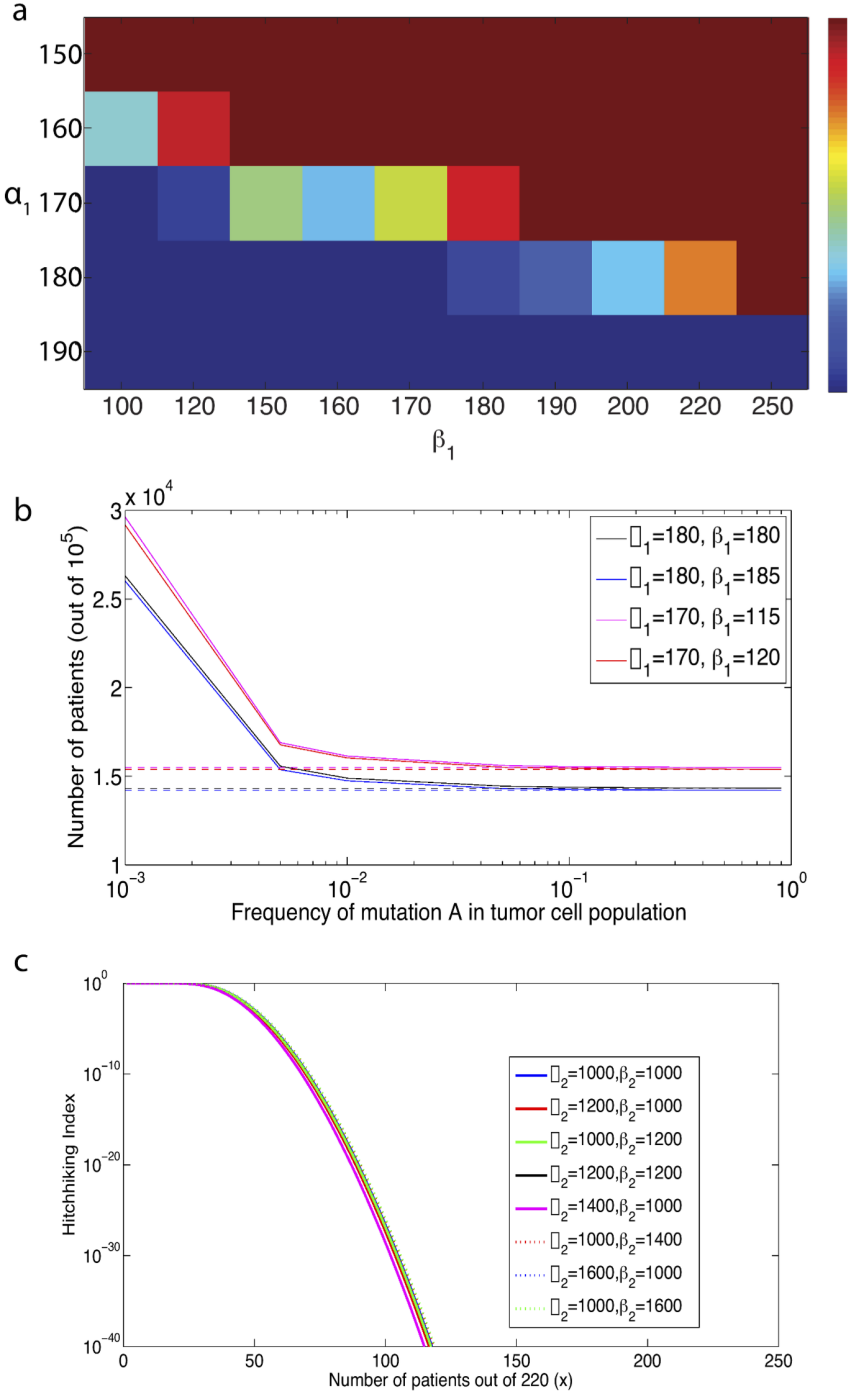
Sensitivity analyses for model predictions. (a) Heatmap for the probability of initiation for a single crypt for ranges of parameters of *α*_1_ and *β*_1_. (b) Number of patients out of 100000 samples in which mutant A cells make up a threshold (x-axis) frequency in the ending tumor cell population, for variable shape parameters of the mutational fitness distribution (*α*_1_,*β*_1_). (Solid) All patients (Dashed) Patients in which mutation A arose and fixed during the pre-initiation phase. (c) Probability of detecting the passenger mutation in x out of 220 patients for a varying *α*_2_, *β*_2_.

We first investigated the sensitivity of our model predictions to the shape parameters of the mutational fitness distribution during the pre-initiation phase of the model. Keeping all other parameters constant, we varied *α*_1_ and *β*_1_. To determine the allowable ranges of these parameters, we first studied the probability of initiation during a lifetime as a function of *α*_1_ and *β*_1_. Note that this is the probability that a single crypt leads to initiation during a lifetime, and there are 15 million crypts in the average human colon. Thus, in order to ensure that the average probability of aberrant crypt foci is between 10 and 90 percent in the population, we require that the probability of initiation from a single crypt is between 0.7×10^−8^ and 6×10^−8^. In Fig 5.a the probability of initiation for a single crypt is plotted for example ranges of parameters *α*_1_,*β*_1_. The intermediate colors (between dark blue and red) represent admissible initiation probabilities. Thus we see that for any given *α*_1_, there is a small range of *β*_1_ that can give rise to the correct range of initiation probabilities. We then investigated the compatibility of these *α*_1_,*β*_1_ combinations with the other incidence data. We found that only *α*_1_ in the range 170 ∼ 180 can give rise to the correct overall lifetime cancer incidence rates within the range 6–10%. Furthermore, for *α*_1_ = 170, *β*_1_ must fall within the narrow range 115 ∼ 120; larger values of *β*_1_ result in lifetime incidence above the allowable range, and lower values of *β*_1_ result in too low an incidence of aberrant crypt foci in the population Similarly, for *α*_1_ = 180, *β*_1_ must lie within the range 180 ∼ 185 to match the incidence data constraints.

Next we used the model to determine the sensitivity of the results, as determined by the Hitchhiking Index, to variations to *α*_1_ and *β*_1_ within these allowable ranges. For example, Fig 5.b shows the number of patients out of 100,000 samples in which cells harboring mutation ‘A’ make up a threshold frequency in the final tumor cell population, for varying *α*_1_ and *β*_1_. We observed only modest differences in *q*, which would translate to negligible differences in the Hitchhiking Index. Therefore, within the constraints of matching the observed incidence data, the Hitchhiking Index is robust to varying the shape parameters of the mutational fitness landscape during the carcinogenesis phase.

### Sensitivity to *α*_2_ and *β*_2_

We also investigated the sensitivity of the results to the shape parameters of the mutational fitness distribution during the clonal expansion phase of the model. Fig 5c. demonstrates the impact of varying *α*_2_ and *β*_2_ up to 60 percent from the original values on the Hitchhiking Index; we found that the Hitchhiking Index is not particularly sensitive to these parameters.

### Sensitivity to initial growth kinetics of clonal expansion, *b* and *d*

We then investigated the model’s sensitivity to the growth rates of the first cell initiating clonal expansion (*b*,*d*). We varied *b* first to determine the impact of the net growth rate on the Hitchhiking Index. Variation of this parameter leads to overall lifetime cancer incidence rates that fall outside the range of our incidence data; this suggests that the net growth rate during the clonal expansion phase within our model should not vary significantly from the fitted value. There is, however, the possibility that both *b* and *d* vary in such a way that the net growth rate remains conserved, for example if both *b* and *d* are increased or decreased by the same amount. These variations might lead to small differences in the model predictions.

## Supporting Information

S1 Dataset The driver gene list. The gene list is based on a Hitchhiking Index of 10^−7^ and is shown together with the output of MutSigCV. Columns: the gene name, the number of unique base pairs that contain non-silent mutations in coding regions, the number of unique base pairs that contain silent mutations in coding regions, the number of non-silent mutations in coding regions, the number of silent mutations, the p-value calculated by MutSigCV, and the q-value calculated by MutSigCV. The gene rank is ordered by q-value from the output of MutSigCV.
(CSV)

S1 Fig Computational analyses of the relationship between the number of identified drivers and the number of samples sequenced. The subsamples consist 10, 20, …, 210 patients, sampled from a total of 220, respectively. The blue line shows the number of identified drivers as a function of number of samples. Error bars are the 95% confidence interval for each subsample. The number of different simulations is 50 for each subsample and the error bar is obtained from the 50 simulations.
(TIFF)
