# Supplementary figures and images for: An Evolutionary Approach for Identifying Driver Mutations in Colorectal Cancer
Source: PLoS Comput Biol. 2015 Sep 17;11(9):e1004350. doi: 10.1371/journal.pcbi.1004350 (PMC4575033; doi:10.1371/journal.pcbi.1004350)

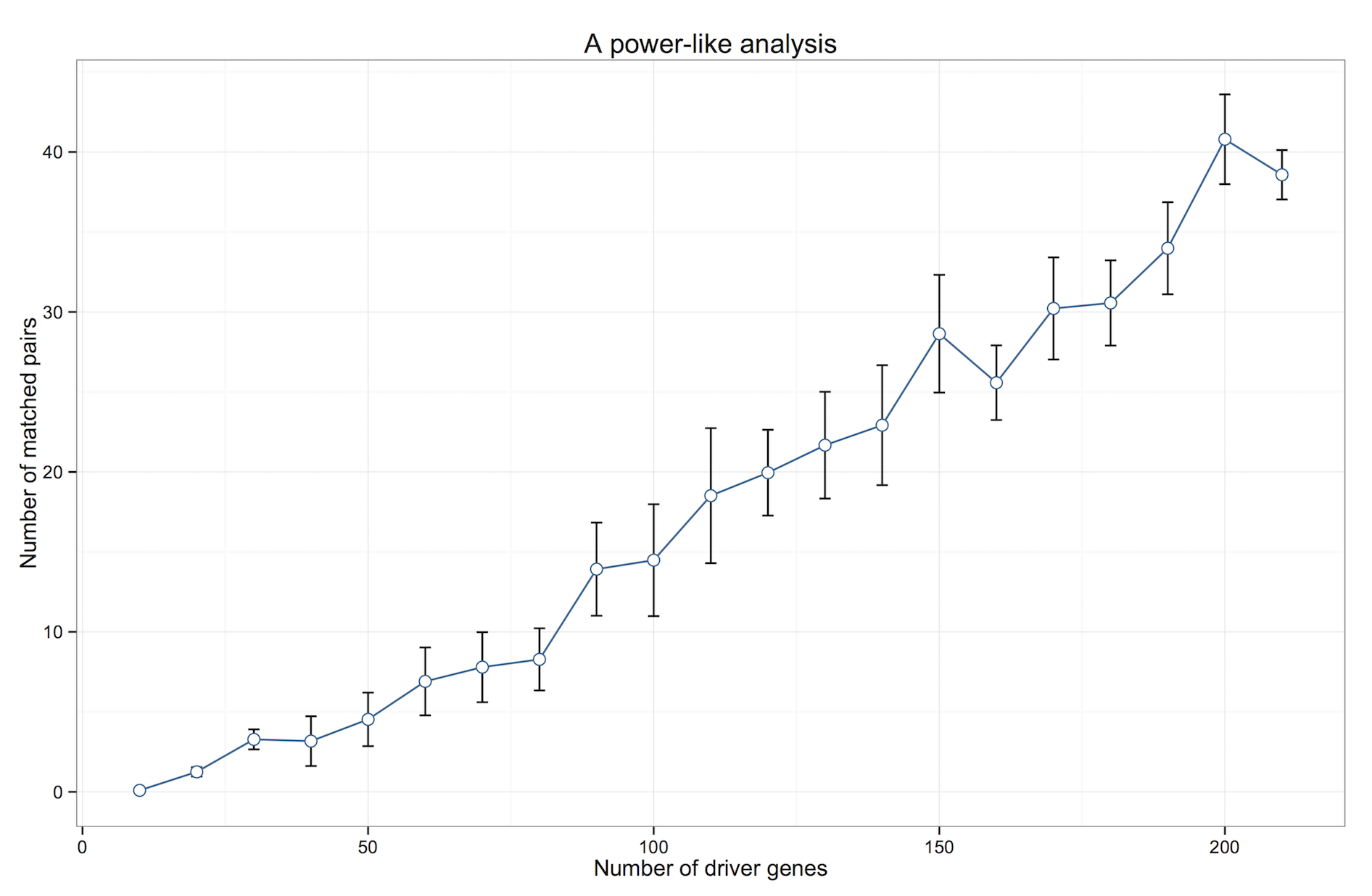

Supplement: S1 Fig — The subsamples consist 10, 20, …, 210 patients, sampled from a total of 220, respectively. The blue line shows the number of identified drivers as a function of number of samples. Error bars are the 95% confidence interval for each subsample. The number of different simulations is 50 for each subsample and the error bar is obtained from the 50 simulations. (TIFF) [file pcbi.1004350.s002.tiff]
